# Changing *Plasmodium falciparum* malaria prevalence in two villages of northeastern Tanzania between 2003 and 2021 in relation to vectors, interventions and climatic factors

**DOI:** 10.1186/s12936-025-05311-y

**Published:** 2025-03-03

**Authors:** Eric Lyimo, Neema B. Kulaya, Lembris Njotto, Nancy A. Kassam, Samwel Gesase, Anangisye Malabeja, Edwin A. Liheluka, Joyce R. Mbwana, Vito Baraka, Michael Alifrangis, Reginald A. Kavishe, Thomas Lavstsen, Bruno P. Mmbando, Thor G. Theander, Daniel T. R. Minja, John P. A. Lusingu, Christian W. Wang

**Affiliations:** 1 National Institute for Medical Research, Tanga Research Centre, Tanga, Tanzania; 2 National Institute for Medical Research, Mwanza Research Centre, Mwanza, Tanzania; 3 Department of Immunology and Microbiology, Centre for Translational Medicine and Parasitology, University of Copenhagen, Copenhagen, Denmark; 4 Kilimanjaro Christian Medical University College (KCMUCo), Moshi, Tanzania; 5 College of Information and Communication Technology, University of Dar Es Salaam, Dar es Salaam, Tanzania; 6 Department of Mathematics and ICT, College of Business Education, Dar es Salaam, Tanzania; 7 Department of Infectious Diseases, Copenhagen University Hospital, Copenhagen, Denmark

**Keywords:** Malaria prevalence, Malaria vector, Bed net, Climatic factors, GSG6-P1

## Abstract

**Background:**

Malaria, which affects over half of the world’s population, is controlled through clinical interventions and vector control strategies. However, these efforts are threatened by resistance to anti-malarial drugs and insecticides, as well as affected by environmental, ecological, and climatic changes. This study examined changes in malaria prevalence and related factors based on data from 18 cross-sectional surveys conducted in two villages in northeastern Tanzania.

**Methods:**

From 2003 to 2021, annual cross-sectional malariometric surveys were conducted in two study villages, Mkokola (lowland) and Kwamasimba (highland), samples collected to determine *Plasmodium falciparum* infection and human exposure to malaria vector *Anopheles*. Pearson's chi-squared test was used for comparing proportions, logistic and linear regressions test were used analyse associations. Generalized Estimating Equations (GEE) was used to analyse the relationship between malaria prevalence and climatic variables.

**Results:**

Malaria prevalence in Kwamasimba and Mkokola dropped from ~ 25% and ~ 80% to 0% and 1%, respectively, between 2003 and 2011, reaching 0% in both villages by 2014. This decline was associated with increased bed net use and reduced exposure to *Anopheles* bites. However, between 2018 and 2021, prevalence resurged, with Kwamasimba reaching 2003–2004 levels despite high bed net use. Between 2003 and 2021 there was an increasing trend in average monthly maximum temperatures (R2 = 0.1253 and 0.2005), and precipitation (R2 = 0.125 and 0.110) as well as minimum relative humidity (R2 = 0.141 and 0.1162) in Kwamasimba and Mkokola villages, respectively, while maximum relative humidity slightly decreased. Furthermore, during 2003–2011, malaria prevalence was positively associated with temperature, maximum temperature, and relative humidity, while precipitation showed a negative association (Estimate:− 0.0005, p < 0.001). Between 2012–2021, all climatic factors, including temperature (Estimate: 0.0256, p < 0.001), maximum temperature (Estimate: 0.0121, p < 0.001), relative humidity (Estimate: 0.00829, p < 0.001), and precipitation (Estimate: 0.000105, p < 0.001), showed positive associations.

**Conclusion:**

From 2003 to 2014, malaria prevalence declined in two Tanzanian villages but resurged after 2018, particularly in highland Kwamasimba. Most likely, vector dynamics affected by changing climatic conditions drove this resurgence, emphasizing the need for adaptive, climate-informed malaria control strategies.

**Supplementary Information:**

The online version contains supplementary material available at 10.1186/s12936-025-05311-y.

## Background

Malaria is a major public health problem that threatens over half of the world's population. In 2022 an estimated 249 million cases resulted in 608,000 deaths with 96% of these deaths occurring in the World Health Organization (WHO) African Region, including 4% in Tanzania [1]. Globally, the incidence of malaria cases decreased by 27% from 2000 to 2019 and then increased by 3% in 2020 and has remained stable. In Tanzania, the estimated annual deaths due to malaria declined from 44,568 to 21,290 from 2000 to 2012 and then increased to 26,334 in 2021 and then again marginally increased to 26,664 in 2022 [1]. These trends reflect the significant progress made in malaria control through dedicated global and regional initiatives, while simultaneously emphasizing the enduring and emerging challenges that pose a threat to sustaining these achievements.

Significant declines in malaria burden are attributed to integrated control measures, including artemisinin-based combination therapy (ACT), insecticide-treated nets (ITNs), indoor residual spraying (IRS), and improved housing [2]. In sub-Saharan Africa, 70% of all households have had at least one ITN by 2022, an increase of 2% in 2021 and roughly an increase of 5% between 2000 and 2022. Despite these achievements, the emergence of resistance to both artemisinin and pyrethroids threatens to undermine these gains [3–6]. In Tanzania and other parts of sub-Saharan Africa, partial resistance to artemisinin has been observed, while pyrethroid resistance, the main class of insecticides used in ITNs, has also been reported [6]. However, ITNs remain more effective than untreated nets in reducing malaria transmission [7].

Malaria transmission is highly sensitive to climatic and environmental factors such as temperature, humidity, and rainfall [8]. Climate change and its consequences such as changes in precipitation pattern and temperature have a significant impact on vector and pathogen dynamics; the changes influences malaria transmission by affecting mosquito breeding, parasite development, and the geographic spread of the disease [8–11].

Ambient temperature significantly impacts malaria dynamics, with rising temperatures accelerating parasite development and shortening the extrinsic incubation period inside *Anopheles* mosquitoes [10, 12]. For instance, an increase in average temperature to around 27 °C causes mosquitoes to take more frequent blood meals and lay more eggs, leading to rapid population growth and increase malaria transmission. [13]. Humidity also influences malaria vectors' lifespan: higher humidity increases their activity and survival, while drier conditions reduce their numbers [14]. Optimal rainfall creates stagnant pools that promote *Anopheles* mosquito propagation, increasing human exposure and hence enhancing malaria transmission while extreme rainfall can disrupt breeding sites [15]. Because of changing and favourable climatic conditions, re-introduction of malaria transmission can be observed in areas that have eradicated malaria but can also facilitate the introduction of malaria in non-endemic areas [16].

Historically, malaria was predominantly found in low altitude regions, however, there has been a notable shift, with the disease now spreading to high altitude areas that were previously unaffected attributed to environmental and vector alterations [17–20]. In high altitude, factors such as changes in rainfall patterns, rising temperature and agricultural practices are believed to play a role in the heightened transmission of malaria in these regions [21]. Warmer temperatures also influence the behaviour and physiology of mosquitoes and responses to warmer and drier conditions among mosquito populations suggest genetic adaptations that could enable their survival and evolutionary response to climate change [22].

The current study was conducted in two rural villages in Korogwe District, Tanga Region, northeastern Tanzania: one in a lowland area (Mkokola) and the other in a highland area (Kwamasimba). Annual malaria surveillance has been ongoing in these villages since 2003, involving blood sampling and recording of age, gender, and bed net usage. Previously, Mmbando et al*.* [23] reported a decline in *P. falciparum* prevalence from 78% in 2003 to 13% in 2008 in the lowland village, and from 25 to 3% in the highland village over the same period.

This study extends that research, aiming to describe changes in malaria point prevalence from 2003 to 2021 in relation to exposure to the malaria vector *Anopheles* spp., bed net use, and changing climatic factors. Since mosquitoes were not collected in the villages, stored blood samples were retrospectively used to assess human exposure to mosquito bites by measuring IgG to mosquito salivary gland proteins. In particular, the *Anopheles gambiae* Salivary Gland protein 6 Peptide 1 (gSG6-P1), as previously done with samples from Kilimanjaro Region, Tanzania [24, 25].

In the study villages as in most of malaria endemic areas, the prevalence is hypothesized to have undergone a significant decline over the study period due to the implementation of control measures. However, a resurgence of malaria cases in recent years, particularly in highland areas like Kwamasimba, is hypothesized to reflect the influence of climatic shifts and evolving vector behaviour, despite the widespread use of bed nets. Retrospective assessments of human exposure to mosquito bites through IgG levels against gSG6-P1 provides additional insights into changes in vector exposure over time.

## Methods

### Study area

Two rural villages, highland Kwamasimba (approximately 700 m above sea level) and lowland Mkokola (approximately 300 m above sea level), were the sites of cross-sectional surveys. The villages are situated in Korogwe District north-eastern Tanzania, approximately 100 kms from Tanga City and the shore of the Indian Ocean. It is a tropical region with two rainy seasons: October to December and April to June. The predominant malaria parasite is *P. falciparum*. Malaria in these two villages is a serious health issue that affects children and adolescents, causing asymptomatic illness and anaemia [26]. Based on a 2018 census survey by the Malaria Research and Capacity building for field trials in Tanzania (MaReCa Project), Kwamasimba and Mkokola villages have human populations of approximately 2000 each.

### Malariometric cross-sectional surveys

From 2003 to 2021, annual malariometric cross-sectional surveys were carried out in the two study villages. However, the year 2020 was skipped owing to restriction imposed by the Tanzanian government due to the COVID-19 pandemic. The surveys were carried out during or after the main rainy season between March and August, and children and adolescents aged between 0 and 19 years old were recruited. The recruitment of study participants was purposely designed to ensure a representative distribution across various age groups. Each year, for both villages the study aimed to include a precise number of individuals within specific age brackets to maintain consistency and reliability in the data collected. The distribution was as follows: 20 participants were recruited from each age groups below 1 year, 2 years, 3 years, 4 years, 5 years, and then from age groups 6 to 7 years, 8 to 9 years, 10 to 11 years, 12 to 13 years, and 14 to 15 years. Additionally, 15 participants were recruited from each of the older age groups, sixteen to seventeen years and eighteen to nineteen years. While the target was set at 250 participants each year, this number was a guideline rather than a strict limit, allowing for some flexibility in recruitment. This structured approach provided a comprehensive and balanced representation of the population across all age categories, ensuring the robustness of the survey findings and facilitating accurate longitudinal analyses over the nearly two-decade span of the study.

Participant’s demographic characteristics were recorded, data on bed net use were also recorded and venous blood was collected. For determining *P. falciparum* infection, blood smear was prepared from whole blood stained and with 10% Giemsa for 15 to 20 min. Two independent microscopists, who were blinded to the malaria rapid diagnostic tests (RDT) results, read the blood smears. In the study area, RDTs were firstly introduced in 2007.

### Evaluation of human IgG antibody levels

Human exposure to mosquito bites was determined from the plasma obtained from the from 13 out of the 18 malariometric cross-sectional surveys from 2004 to 2008, 2012, and from 2014 to 2019 and lastly 2021. During cross-sectional surveys, plasma samples were collected from venous or finger prick EDTA blood by spinning the vacutainers at 2000 RPM for 10 min and aliquots were kept at − 20 °C until further analyses. Plasma samples were available for 1296 and 1266 individuals from Kwamasimba and Mkokola villages, respectively (Additional Table 1). Lyophilized synthetic gSG6-P1 antigen, as previously described [27] was obtained from Genepep SA (St-Jean de Vedas, France). The lyophilized gSG6-P1 antigen was then dissolved to a final working concentration of 20 μg/mL in ultra-filtered water.

**Table 1:** Characteristics of the study population from Kwamasimba and Mkokola villages, northeastern, Tanzania

		Kwamasimba (n = 4915)	Mkokola (n = 4921)
Gender, n (%)	Female	2671 (54.4)	2587 (52.7)
	Male	2236 (45.6)	2325 (47.3)
Malaria prevalence by blood smear, n (%)	Positive	313 (6.4)	1036 (21.2)
	Negative	4578 (93.6)	3853 (78.8)
Bed net use, n (%)	No	1711 (35.0)	1161 (23.6)
	Yes	3183 (65.0)	3749 (76.4)
Age groups, n (%)	0–4	1929 (39.2)	1859 (37.8)
	5–9	1427 (29.0)	1542 (31.3)
	10–14	1081 (22.0)	1043 (21.2)
	15–19	478 (9.7)	477 (9.7)
Weight (Kg)	Median [IQR]	18 [12, 28]	19 [13.0, 29.1]
Year of survey, n sampled (%)	2003	283 (5.8)	309 (6.3)
	2004	259 (5.3)	257 (5.2)
	2005	273 (5.6)	324 (6.6)
	2006	254 (5.2)	302 (6.1)
	2007	291 (5.9)	304 (6.2)
	2008	235 (4.8)	227 (4.6)
	2009	265 (5.4)	238 (4.8)
	2010	275 (5.6)	284 (5.8)
	2011	263 (5.4)	272 (5.5)
	2012	287 (5.8)	259 (5.3)
	2013	275 (5.6)	270 (5.5)
	2014	274 (5.6)	271 (5.5)
	2015	282 (5.7)	293 (6.0)
	2016	277 (5.6)	262 (5.3)
	2017	300 (6.1)	269 (5.5)
	2018	280 (5.7)	285 (5.8)
	2019	275 (5.6)	261 (5.3)
	2021	267 (5.4)	234 (4.8)

The Enzyme-Linked Immuno-Sorbent Assay (ELISA) method was used, as previously described [24, 28]. Briefly, 100 μL/well of 20 μg/mL gSG6-P1 antigen was coated onto 96-well micro-assay plates (Maxisorp in Roskilde, Denmark) and incubated at 37 °C for 2 h 30 min. Plate wells were then blocked for 1 h with 100 μL of 0.5% Casein containing 0.05% Tween20. 20% test sera, diluted in blocking buffer, were added and incubated at 2 to 8 °C overnight. After washing anti-gSG6-P1 IgG was detected with 100 μL/well horse radish peroxidase (HRP)-conjugated goat anti-human IgG antibody (Thermo Fisher Scientific) diluted 1:10,000 in PBS and incubated at 22 °C for 1 h. Later, each well received 100 μL/well of 2,2′-azino-bis (3-ethylbenzothiazoline-6-sulfonic acid) (ABTS) (Roche, Germany) and incubated for 50 min at 22 °C and to stop the enzymatic reaction, wells were incubated with 10 μL of 20% sodium dodecyl sulfate (SDS) (SigmaAldrich) solution. Optical density (OD) measurements were read at 405 nm on Multi-Scan FC microplate photometer (Thermo Scientific, Life Technologies Corporation) ELISA reader. The mean OD of ten Danish volunteers’ unexposed controls plus two standard deviations were used to calculate the cut-offs for seropositivity for each plate. A pool of eight positive control plasma sample taken from confirmed cases of malaria was included in each run for quality control as cut-offs were established for each run.

### Total precipitation, temperature, and relative humidity

The monthly total precipitation, daily 2-m air temperature and daily dew point temperature data from 2003 to 2021 were retrieved from the ERA5 Copernicus Climate data store [29]. The 2 m dew point parameter represents the temperature to which the air, at a height of 2 m above the Earth's surface, must be cooled to reach saturation. It serves as an indicator of air humidity. When combined with 2 m air temperature, it can be used to calculate relative humidity. ERA5 reanalysis employs the Integrated Forecasting System (IFS) to simulate past weather and climate conditions. Precipitation in the European Centre for Medium-Range Weather Forecasts IFS is generated by the cloud scheme. This scheme models the formation and dissipation of clouds, as well as large-scale precipitation, based on changes in atmospheric variables such as pressure, temperature, and moisture, predicted directly by the IFS at the grid box scale or larger. Convective precipitation is generated by the convection scheme within the IFS, which represents convection occurring at spatial scales smaller than the grid box. Precipitation is measured in units of depth in metres of water equivalent. This represents the depth the water would have if it were evenly distributed over the grid box. Monthly average minimum and maximum 2 m air temperatures and relative humidity were calculated from the retrieved daily maximums and minimums. The relative humidity was calculated from air temperature and dew point temperature data using the online relative humidity calculator [30].

### Statistical analysis

Data were double entered into a Microsoft Access database, cleaned, and processed in Microsoft Excel (Microsoft Corp., Redmond, WA, USA) and transferred into STATA version 15.0 (StataCorp, TX, USA) and for statistical analyses, figures and analyses were drawn and done with R version 4.3.1, respectively. Pearson's chi-squared test was used to test proportions of variables between the two villages and years of surveys. A nonparametric equality-of-medians test was used to compare the median values for anti-gSG6-P1 ODs between the two villages in each of the thirteen cross-sectional surveys to assess which villages had more exposure to *Anopheles* bites. Logistic regression was employed to analyse *P. falciparum* prevalence, while linear regression was utilized to assess seasonal variation of anti-gSG6-P1 antibodies. Both analyses were adjusted for gender, bed net use, and age groups. Simple linear regression was used to assess climatic variables trends from 2003 to 2021. Generalized Estimating Equations (GEE) approach was used to analyse the relationship between malaria prevalence and climatic variables in the study area from 2003 to 2021. The dependent variable was annual malaria prevalence, while independent variables included annual average temperature, maximum temperature, relative humidity, and total precipitation; minimum temperature was excluded due to multicollinearity. Missing prevalence data for 2020 were imputed using linear interpolation. The GEE model employed an exchangeable correlation structure and a Gaussian identity link function to estimate marginal effects, focusing on two trends: a decline from 2003 to 2011 and an increase from 2012 to 2021. For all the statistical tests results were considered statistically significant at p < 0.05.

## Results

### Study population and demographic characteristics

During the 18 annual malariometric cross-sectional surveys, a total of 4915 and 4921 children and adolescents between the age of 0 and 19 years were recruited in the two villages Kwamasimba and Mkokola, respectively (Table 1). The gender distribution in Kwamasimba and Mkokola were females and males: 54.4 and 45.6, and 52.7 and 47.3%, respectively.

### Malaria point prevalence in Kwamasimba and Mkokola villages from 2003 to 2021

**Fig. 1:**
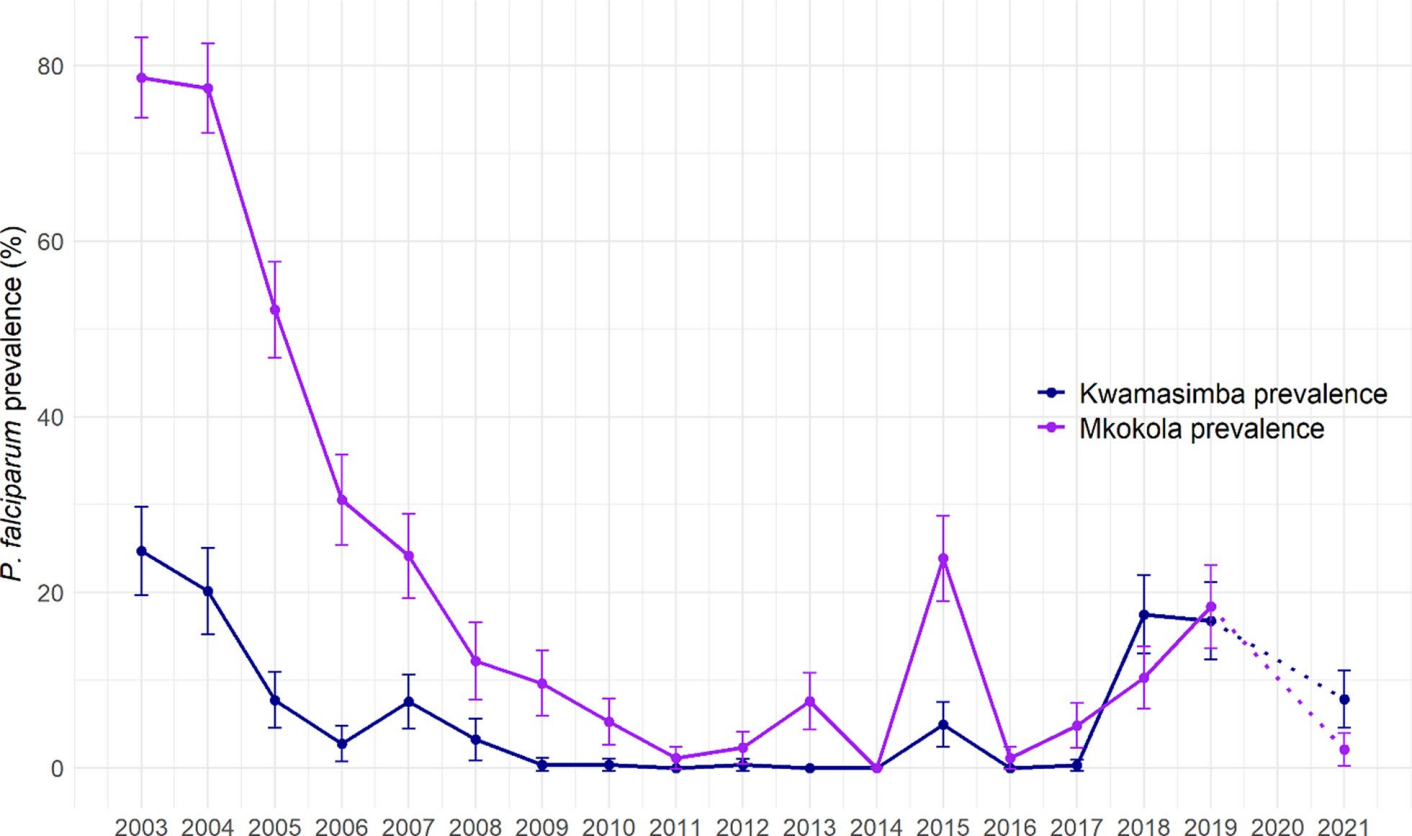
*Plasmodium falciparum* point prevalence (with 95% CI) in highland Kwamasimba and lowland Mkokola from north-eastern Tanzania. The 18 annual malariometric cross-sectional surveys were conducted over a 19 year period from 2003 to 2021, with the exception of 2020 due to COVID-19 pandemic restrictions. A total of 4915 and 4921 children and adolescents between the age of 0 and 19 were recruited in the Kwamasimba and Mkokola, respectively

The eighteen malariometric cross-sectional surveys were conducted annually during high transmission season from 2003–2019 and in 2021. In highland Kwamasimba in 2003, malaria point prevalence was 24.7% which decreased progressively to 0% in 2011 (Fig. 1). From 2012 to 2017 the malaria prevalence was between 0–0.4% with the exception of 2015 showing a peak of 5% prevalence. In 2018, malaria point prevalence increased significantly (χ^2^ = 54.179, *p* < 0.001) to 17.5%, 16.8% in 2019, and decreased to 7.9% in 2021.

The malaria point prevalence in lowland Mkokola in 2003 was 78.6% and significantly higher than in Kwamasimba (χ^2^ = 172.262, *p* < 0.001). From 2004 the prevalence decreased progressively from 77.4% to 1.1% in 2011. From 2011 to 2017 the malaria prevalence was between 2.3–7.6% with the exception of year 2015 showing a peak of 23.9%. In 2018, malaria point prevalence increased to 10.3%, then to 18.4% in 2019, and decreased to 2.1% in 2021. In the years 2018 and 2021, a significantly higher malaria prevalence was reported in highland Kwamasimba than in lowland Mkokola (χ^2^ = 6.121, *p* = 0.013 and χ^2^ = 8.317, *p* = 0.004, respectively).

### Bed net use

**Fig. 2:**
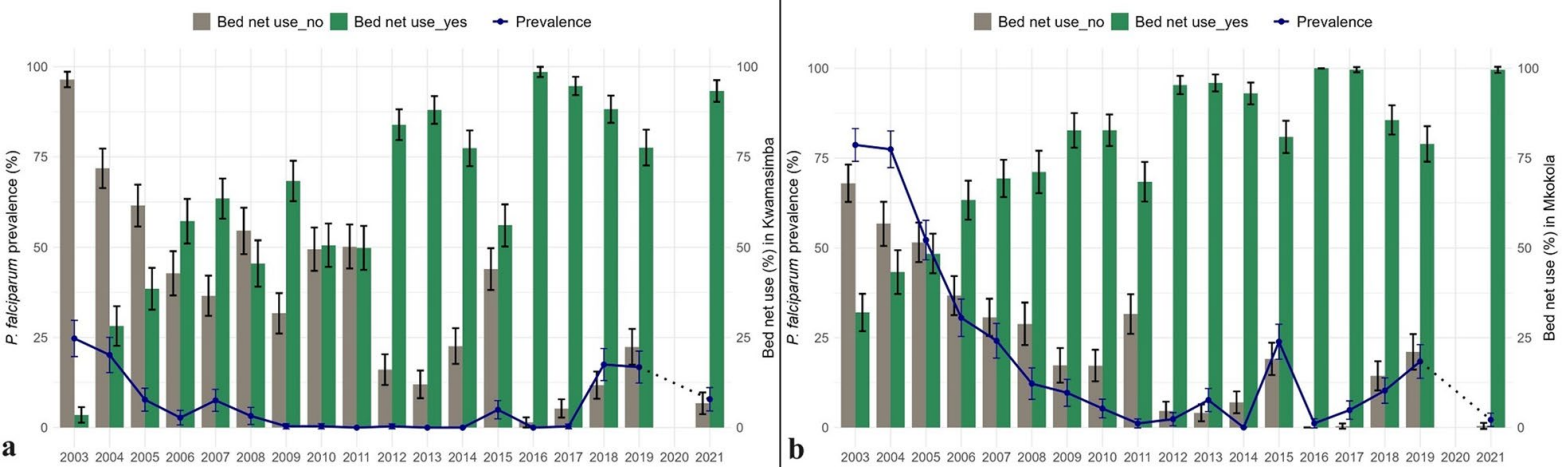
Bed net use and *P. falciparum* prevalence in (**a**) highland Kwamasimba, (**b**) lowland Mkokola. The study population included in all 18 malariometric cross-sectional surveys consisted of 4915 individuals from Kwamasimba and 4921 individuals from Mkokola

In Kwamasimba, the bed net use was only 3% in 2003 but increased to more than 80% in 2012 and remained around 70% or above, with the exception of a significant decrease to 56% in 2015 from 77% in 2014 (χ2 = 28.439, p < 0.001) (Fig. 2a and Additional Table 2). Additionally, there were significant drops in bed net use from 94.8% in 2017 to 88.2% in 2018 (χ2 = 7.796, *p* = 0.005) and then to 77.6% in 2019 (χ2 = 11.057, *p* < 0.001). In Mkokola, bed net use increased from around 30% in 2003 to more than 80% in 2009 and remained around 80% or above, with the exception of 2011, where a decrease to 68% was reported (Fig. 2b and Additional Table 2). A significant drop in bed net use from 93.0% to 80.9% between 2014 and 2015 was also observed in Mkokola (χ2 = 17.882, *p* < 0.001). Further, significant decreases were observed in Mkokola from 99.6 to 85.6% between 2017 and 2018 (χ2 = 38.791, *p* < 0.001) and then to 78.9% in 2019 (χ2 = 4.204, *p* = 0.040). Between 2003 and 2015, bed net use was generally higher in lowland Mkokola than in highland Kwamasimba, and comparable from 2016 and onwards.

**Table 2:** Univariate and multivariate logistic regression analysis of the association between *P. falciparum* point prevalence with bed net use, and age groups in the Kwamasimba highland village from 2003 to 2021

	COR (95% conf. interval)	p-value		AOR (95% conf. interval]	Adjusted p-value	Overall adjusted p-value
Gender						
Female	Ref.			Ref.		0.001*
Male	1.324 (1.053–1.665)	0.016*		1.362 (1.078–1.721)	0.010*	
Bed net use						
No	Ref.	Ref.		Ref.		
Yes	0.326 (0.258–0.413)	0.000*		0.336 (0.265–0.426)	0.000*	
Age groups						
0–4	Ref.		0.0018	Ref.		
5–9	1.177 (0.874–1.585)	0.282		1.175 (0.868–1.590)	0.297	
10–14	1.533 (1.134–2.073)	0.006*		1.505 (1.106–2.047)	0.009*	
15–19	1.924 (1.332–2.779)	0.001*		1.801 (1.238–2.621)	0.002*	

AOR: Adjusted Odds Ratio; COR: Crude Odds Ratio; Ref.: Reference

^*^Statistical significance

### Trends in exposure to *Anopheles* mosquito bites

**Fig. 3:**
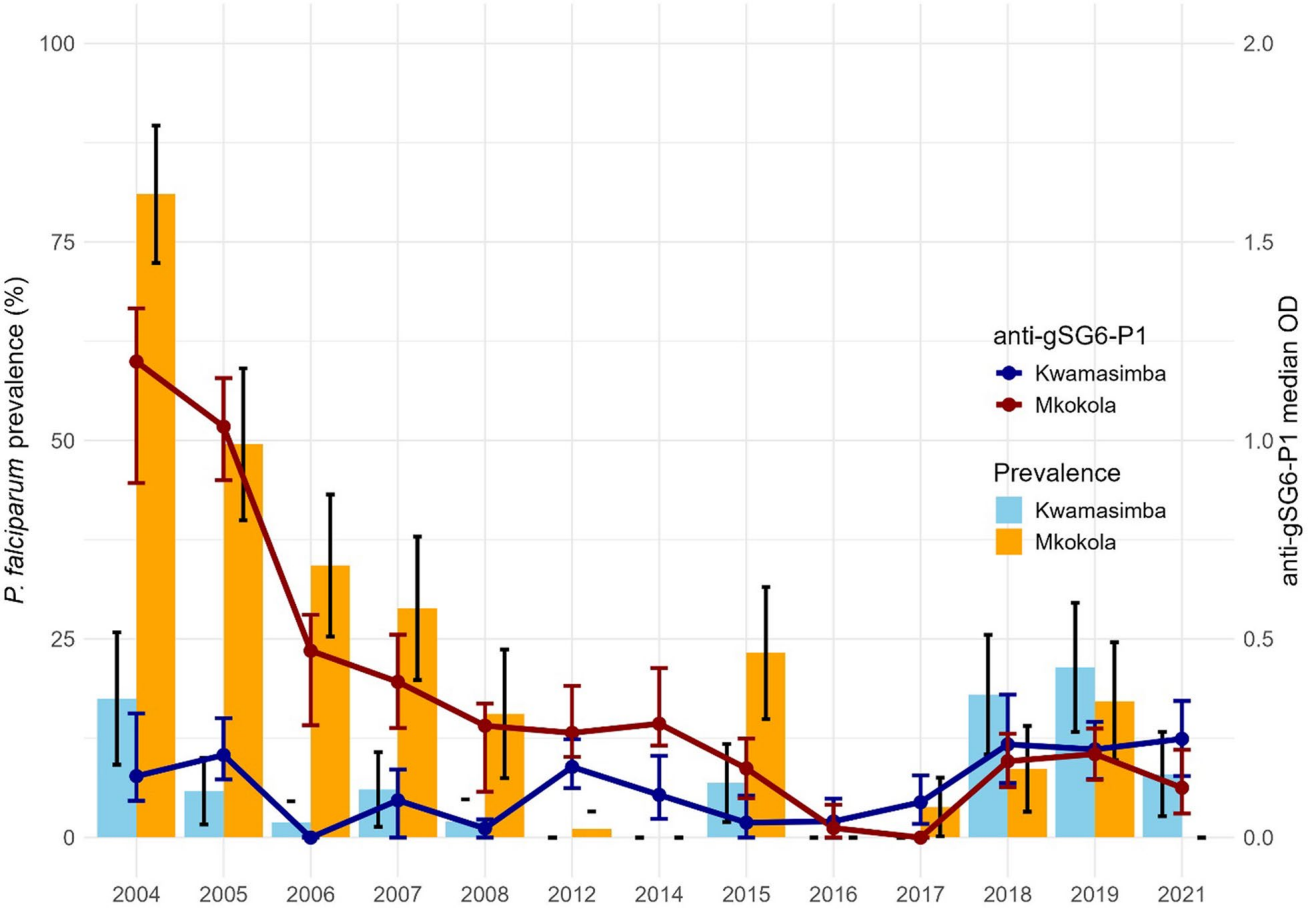
Anti-gSG6-P1 median OD and *P. falciparum* prevalence in Kwamasimba and Mkokola. A sub-population from 13 out of the 18 cross-sectional surveys was investigated for *Anopheles* bite exposure, with sample sizes of 1296 for Kwamasimba and 1266 for Mkokola

Antibodies to the mosquito salivary gland protein gSG6-P1 were used as a biomarker to determine the levels of exposure to *Anopheles* in the two villages. Plasma samples were available from 13 out of the 18 cross-sectional surveys starting from 2004 (Additional Table 2B). In Mkokola, antibody levels progressively decreased from 2004 to 2017, then increased significantly in 2018 (χ^2^ = 26.327, p < 0.001). This increase remained steady in 2019 and 2021 (Fig. 3). However, the antibody levels were still significantly lower compared to the period from 2004 to 2007 (χ^2^ = 8.359, p = 0.004). In Kwamasimba in 2004, the participants had significantly lower antibody levels than the participants in Mkokola (χ^2^ = 47.601, *p* < 0.001). The antibody levels in Kwamasimba decreased from 2005, reached nearly zero in 2006 and 2008, then significantly increased in 2018 (χ2 = 5.780, p < 0.001) and remained stable at this higher level through 2021, similar to the levels observed in 2004 and 2005. A peak in antibody levels was also seen in 2012 in Kwamasimba. The antibody levels were higher in Mkokola than in Kwamasimba from 2004 until the 2016 cross-sectional survey (χ^2^ = 0.700, *p* = 0.403), while in 2017 the levels were significantly higher in Kwamasimba (χ^2^ = 13.260, *p* < 0.001) and at same level in 2018 and 2019 (χ^2^ = 0.079, *p* = 0.779 and χ^2^ = 0.127, *p* = 0.722, respectively). In 2021, the antibody level in Kwamasimba was again significantly higher than in Mkokola (χ^2^ = 4.228, *p* = 0.04).

### Correlation between malaria point prevalence and anti-gSG6-P1 antibody levels

The correlation between malaria point prevalence from each cross sectional survey and the anti-gSG6-P1 OD median values were analysed by Spearman’s rank correlation test. The point prevalence represents the proportion of individuals in a given cross-sectional survey, and anti-gSG6-P1 OD median values shows the level of exposure to *Anopheles* spp. mosquito bites in that survey. There was a strong correlation between malaria point prevalence and anti-gSG6-P1 antibody levels in Kwamasimba and Mkokola (Spearman’s rho = 0.6, *p* < 0.001) (Additional Fig. 1).

### The association of malaria prevalence and *Anopheles* exposure, gender, bed net use and age

Uni- and multivariate logistic regression was used to explore the association between gender, bed net use ´age and malaria prevalence (Tables 2 and 3). Males were significantly more likely to test positive in both Kwamasimba (AOR 1.362,95% CI 1.078–1.721, p = 0.010) and Mkokola (AOR 1.214, 95% CI 1.049–1.406, p = 0.009) (Tables 2 and 3). Bed net use was associated with a lower risk of being malaria positive in both Kwamasimba and Mkokola (respectively, AOR 0.336, 95% CI 0.265–0.426, *p* < 0.001 and AOR 0.202, 95% CI 0.174–0.235, *p* < 0.001). In Kwamasimba, participants above 10 years old were significantly more likely to test positive for malaria than those who were younger; age group 10–14 (AOR 1.505, 95% CI 1.106–2.047, p = 0.009) and age groups 15–19 (AOR 1.801, 95% CI 1.238–2.621, p = 0.002), respectively. In Mkokola, participants below 10 years were more likely to be malaria-positive than those between 10 to 19 years old, but the association was not statistically significant (Table 3).

**Table 3:** Univariate and multivariate logistic regression analysis of the association between *P. falciparum* prevalence with bed net use, and age groups in the Mkokola lowland village from 2003 to 2021

	COR (95% conf. interval)	p-value		AOR (95% conf. interval]	Adjusted p-value	Overall adjusted p-*value*
Gender						
Female	Ref.	Ref.		Ref.	Ref.	0.001
Male	1.208 (1.053–1.386)	0.007*		1.214 (1.049–1.406)	0.009*	
Bed net use						
No	Ref.	Ref.		Ref.	Ref.	
Yes	0.205 (0.177–0.238)	0.001*		0.202 (0.174–0.235)	0.001*	
Age groups						
0–4	Ref.	Ref.		Ref.	Ref.	
5–9	1.084 (0.920–1.277)	0.333	0.247	1.039 (0.873–1.235)	0.670	
10–14	0.930 (0.770–1.123)	0.450		0.913 (0.747–1.116)	0.375	
15–19	0.867 (0.672–1.120)	0.274		0.768 (0.586–1.007)	0.057	

AOR: Adjusted Odds Ratio; COR: Crude Odds Ratio; Ref.: Reference

^*^Statistical significance

### The association of exposure to *Anopheles* mosquito bite and gender, bed net use and age

The association of exposure to *Anopheles* mosquito bites and gender, bed net use, and age were explored by linear regression (Additional Table 3). Bed net use was associated with lower antibody levels in both Kwamasimba (adjusted coef − 0.073, CI: − 0.123–− 0.022, *p* = 0.005) (Additional Table 3), and Mkokola (adjusted coef − 0.330, CI − 0.403–− 0.256, p < 0.001) (Additional Table 4). Age above four years of age was associated with higher antibody levels in both villages (Additional Table 3 and 4). Seasonal trend in temperature, precipitation, and relative humidity from 2003 to 2021.

**Fig. 4:**
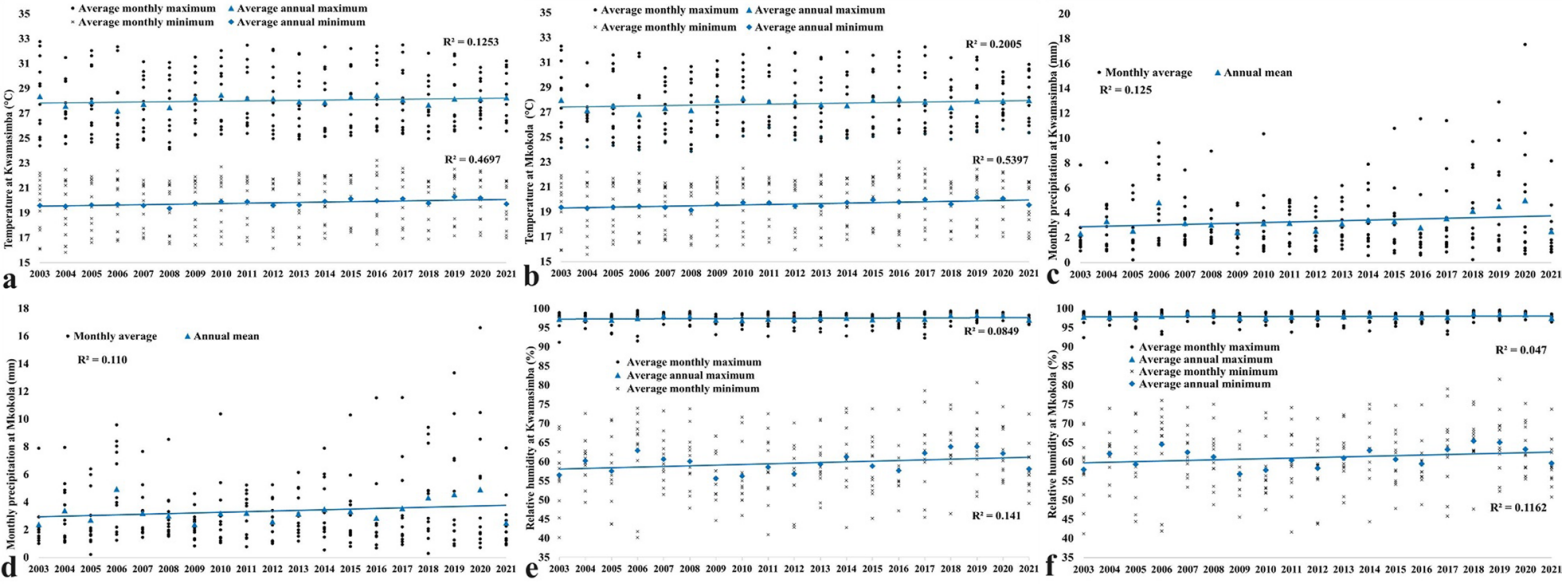
Climatic data from Kwamasimba and Mkokola villages from north-eastern Tanzania 2003 to 2021. Average monthly minimum and maximum temperature in (**a**) Kwamasimba and (**b**) Mkokola, average monthly precipitation in (**c**) Kwamasimba and (**d**) Mkokola, average monthly minimum and maximum relative humidity in (**e**) Kwamasimba and (**f**) Mkokola

From 2003 to 2021 the average monthly minimum temperature showed an increase for both highland Kwamasimba (R2 = 0.4697) and lowland Mkokola (R2 = 0.5397), whereas the average monthly maximum temperature showed only a small positive trend (Kwamasimba; R2 = 0.1253 and Mkokola; R2 = 0.2005, respectively) (Figs. 4a and b). There was a slightly positive trend in the average monthly precipitation in both Kwamasimba (R^2^ = 0.125) and Mkokola (R^2^ = 0.110) (Figs. 4c and d) as well as a slightly positive trend in the average monthly minimum relative humidity; Kwamasimba (R2 = 0.141) and Mkokola (R2 = 0.1162), respectively. The average monthly maximum relative humidity, however showed a small negative trend (R2 = 0.0849) and (R2 = 0.047) in Kwamasimba and Mkokola (Figs. 4e and f).

### Temporal associations between climatic factors and malaria prevalence

**Fig. 5:**
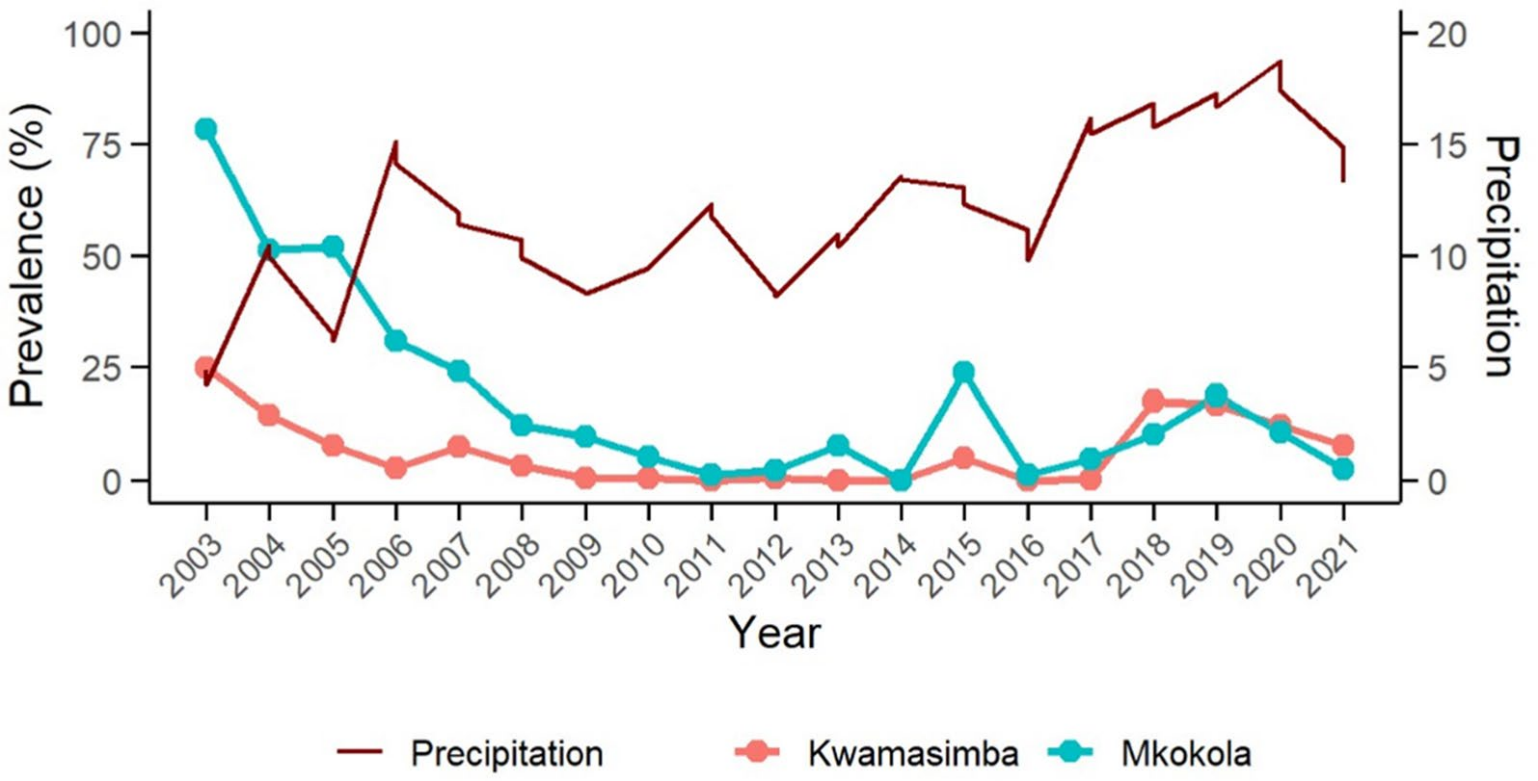
The association between prevalence and precipitation in Kwamasimba and Mkokola villages from north-eastern Tanzania 2003 to 2021. There was a decreasing trend of precipitation and prevalence from 2003 to 2011 and an increasing trend from 2012 to 2021

The GEE analysis showed distinct trends in malaria prevalence and its associations with climatic variables across two time periods between 2003–2011 and 2012–2021. During 2003–2011, malaria prevalence exhibited a decreasing trend and there were significant positive associations with annual average temperature (Estimate: 0.123, p < 0.001), maximum temperature (Estimate: 0.026, p < 0.001), and relative humidity (Estimate: 0.04, p < 0.001). In contrast, total precipitation was negatively associated with prevalence (Estimate: − 0.0005, p < 0.001) (Fig. 5). During 2012–2021, prevalence followed an increasing trend, and all climatic factors showed positive associations, including annual average temperature (Estimate: 0.0256, p < 0.001), maximum temperature (Estimate: 0.0121, p < 0.001), relative humidity (Estimate: 0.00829, p < 0.001), and total precipitation (Estimate: 0.000105, p < 0.001) (Fig. 5). The magnitude of the effects for temperature and humidity was lower in the latter period, while the direction of the precipitation effect shifted from negative to positive, reflecting dynamic changes in the relationships between climatic factors and malaria prevalence.

## Discussion

In this study, the malaria point prevalence in two rural villages of north-eastern Tanzania was investigated from 2003 to 2021. Two similar villages in size and demographics only 15 km apart but situated in areas historically of markedly different transmission intensities: high malaria transmission lowland Mkokola and low malaria transmission highland Kwamasimba [23, 31]. The malaria point prevalence decreased from ~ 25% and ~ 80% to 0% and 1% in Kwamasimba and Mkokola, respectively, from 2003 to 2011 and fell to 0% in both villages in 2014, following the trend in malaria decline in the entire malaria endemic regions within Africa; partially in response to the increased efforts in prevention, prompt diagnosis, and treatment with effective anti-malarial drugs [32–34], though these factors do not fully account for the observed changes [35]. A similar decline in malaria prevalence in the same period of time in Muheza District [11], adjacent to Korogwe District, has been partly attributed to changes in the composition and a significant reduction in the abundance of malaria vectors, *Anopheles* spp., in the region [36, 37].

Bed net use has been strongly associated with a reduction in malaria prevalence as it corresponds with the prevention of *Anopheles* infectious bites and bed net usage in Kwamasimba and Mkokola increased significantly as Tanzania expanded the availability and accessibility of long-lasting insecticide-treated bed nets to pregnant women (starting in 2004) and infants (starting in 2006) [38, 39] and the malaria prevalence decreased concordantly with the exposure to *Anopheles* mosquito bites. Antibodies to mosquito salivary gland proteins were used to assess historical exposure to mosquitoes by analysing archived plasma samples, as entomological data were unavailable for the study period. This method has previously proven to be a useful tool for detecting and distinguishing temporal and spatial variations in exposure to mosquito bites [24, 25], and this current study further validates and extend the use.

The observed decrease in *P. falciparum* prevalence in the two villages began before Tanzania adopted ACT as the first-line treatment and introduced in the village in 2007 [23]. A shift was seen from 2017 where the exposure to mosquitoes increased and was higher in highland Kwamasimba than in lowland Mkokola despite continuous high bed net use > 77% in both villages, bed nets are effective in blocking malaria transmission when usage exceeds 70% [40]. Interestingly, besides a temporary spike in malaria prevalence in 2015, malaria prevalence also significantly increased in 2018–2019, with Kwamasimba showing a resurgence to the high levels reported in 2003–2004. For the first time since surveillance began, Kwamasimba had higher malaria prevalence than lowland Mkokola. The resurgence in this period also coincided with a significant drop in bed net use between 2017–2019 in both villages, though still above 70% [40]. The resurgence in malaria prevalence between 2018–2019, despite sustained bed net coverage (> 77%), likely reflects pyrethroid resistance in local mosquito populations. While the current study did not assess vector resistance, regional study demonstrates that by 2020, over 40% of *An. gambiae *sensu lato in Tanzania survived pyrethroid exposure [6]. This resistance may have diminished ITN efficacy, even as coverage remained high, concurrently, climatic shifts such as rising temperatures and outdoor tendencies of *Anopheles arabiensis* likely compounded transmission risk [10, 41, 42]. The variability in bed net usage, coupled with the rising incidence of insecticide resistance, may have contributed to the heightened mosquito exposure and the resurgence of malaria in both villages. This underscores the critical importance of maintaining consistent and effective vector control strategies.

Climatic factors may have also contributed to the resurgence in malaria vector exposure and malaria cases with different implications for relatively cooler highland vs. warmer lowland areas [9]. Temperature, precipitation, and humidity are important factors that greatly influence the transmission dynamics of malaria [22]. Even small differences can create more favourable conditions for mosquito survival and breeding areas that could not support vector existence before, thereby increasing the likelihood of malaria transmission [8, 43].

Temperature is crucial, with transmission intensity peaking at around 27 °C and optimal vector survival between 17 °C and 32 °C [44]. In both villages, temperatures fall within this ideal range for most months of the year. In the period of 2003 to 2021 the annual monthly average maximum temperature was between 27.2–28.5 °C and 26.8–26.8 °C, and annual monthly minimum average temperature was between 19.4–20.3 °C and 19.1–20.2 °C for Kwamasimba and Mkokola, respectively. Increasing temperatures and precipitation may have enhanced mosquito survival and breeding, during the phase of increasing malaria prevalence trends all climatic factors were positively associated with prevalence. Interestingly, during the phase of decreasing malaria prevalence there was a positive association of temperature and relative humidity with prevalence suggesting that these factors created favourable conditions for mosquito vectors. Nevertheless, the effects of the temperature and relative humidity were outweighed with effectiveness of intensified malaria control interventions such as insecticide-treated nets, indoor residual spraying and improved treatment availability. The positive association and increasing trend of climatic factors likely associated with enhanced mosquito survival and breeding, may have contributed to resurgence in malaria prevalence, particularly in highland Kwamasimba, as observed elsewhere [43, 45]. Notably, the association between malaria prevalence and precipitation shifted from negative to positive in recent surveys. These findings highlight the dynamic interplay between climate, intervention strategies, and environmental changes in shaping malaria prevalence.

In 2012, it was reported that once the most abundant sibling species, *An. gambiae s.s.*, had become the rarest, while *An. arabiensis* had shifted from being the rarest to the most common around the years 2006–2011 in the same region [36]. While transmission by the more anthropophilic and endophilic *An. gambiae *sensu stricto (*s.s*.) [46, 47] is declining, it is being replaced by the more adaptable *An. arabiensis* [46, 48]. Although *An. arabiensis* is thought of as a relatively poor vector and exhibits zoophilic and exophilic behaviour [49], the observed increase in malaria prevalence and *Anopheles* mosquito bites combined with the relatively high bed net use in our settings suggests it may have adapted to become the predominant malaria vector species, as also shown in a recent publication of the national malaria vector entomological surveillance in mainland Tanzania from 2017–2021 [50], highlighting the requirement of tools addressing outdoor transmission mediated by *An. arabiensis*.

This study lacks entomological data, such as vector species composition and identification, which limits comprehensive interpretation of the exact *Anopheles* species responsible for transmission in the study area. While IgG antibodies to *Anopheles* salivary gland proteins (gSG6-P1) provide valuable insights into human exposure to bites [24, 25], this approach cannot resolve species-specific shifts or behavioural adaptations. For instance, the replacement of *An. gambiae s.s.* by *An. arabiensis*, a species with distinct zoophilic feeding and outdoor resting behaviours [46, 49], could reduce the efficacy of indoor-focused interventions like ITNs and IRS, thereby sustaining mosquito exposure [50], and causing antibody levels to remain stable. The retrospective design of the current study reliant on archived blood samples, precluded concurrent entomological monitoring [27, 51, 52].

Nevertheless, the gSG6-P1 biomarker remains a functional proxy for cumulative *Anopheles* exposure and complements clinical malaria trends [51]. Future studies combining serological assays with entomological surveillance could clarify how vector shifts interact with climate and interventions to shape malaria risk. While this study is limited to two villages in northeastern Tanzania, the findings offer insights into climate driven malaria trends and vector shifts relevant to similar endemic regions. However, ecological and intervention differences may affect generalizability, warranting future multi-site comparisons for broader validation.

Demographic factors further underscore the complexity of malaria risk in these communities. Males were more likely to test positive for malaria than females, a pattern potentially explained by greater male engagement in outdoor activities [53, 54] and lower bed net usage among males compared to females [55, 56]. Similarly, increasing age in highland Kwamasimba correlated with heightened exposure to *Anopheles* bites, a trend observed in other settings [57, 58], which may be due to older individuals perceived the risk of malaria as low and, therefore, will tend not to use protective measures against mosquito bites [59, 60].

These findings highlight how behavioural and sociodemographic factors intersect with ecological and climatic drivers to shape malaria transmission. Addressing these dynamics through community education targeting all age groups, and adaptive strategies that account for outdoor exposure will be critical to sustaining progress in malaria control amid shifting transmission patterns.

## Conclusion

From 2003 to 2021, malaria prevalence in two rural villages in north-eastern Tanzania significantly decreased, but since 2018, malaria cases have resurged, particularly in highland Kwamasimba, despite high bed net use. This resurgence may be partly attributed to climatic factors, with changes in temperature, humidity, and precipitation positively influencing transmission and potentially outweighing the declining efficacy of control interventions. These findings highlight the need for continuous monitoring and adaptive malaria control strategies, especially also considering changes in climate and vector behaviour.

## Supplementary Information

Supplementary Material 1

## Data Availability

The datasets used and analysed in this study are available from the corresponding author (EL) upon reasonable request per institutional guidelines and permission from the Medical Research Coordinating Committee (MRCC) of Tanzania.

## Abbreviations

ACT: Artemisinin-based combination therapy
COVID-19: Coronavirus Disease 2019
ELISA: Enzyme-Linked Immuno-Sorbent Assay
GEE: Generalized Estimating Equations
gSG6-P1: *gambiae* Salivary gland protein 6 peptide 1
HRP: Horse radish peroxidase
IPT: Intermittent preventative treatment
IRS: Indoor residual spraying
ITNs: Insecticide-treated nets
RDT: Malaria rapid diagnostic tests
OD: Optical density
SDS: Sodium dodecyl sulfate
WHO: World Health Organization
