# Supplementary material for: Changing Plasmodium falciparum malaria prevalence in two villages of northeastern Tanzania between 2003 and 2021 in relation to vectors, interventions and climatic factors
Source: Malar J. 2025 Mar 3;24:68. doi: 10.1186/s12936-025-05311-y (PMC11874435; doi:10.1186/s12936-025-05311-y)
Supplement: Supplementary file 1 — Supplementary Material 1 [file 12936_2025_5311_MOESM1_ESM.docx]

**Additional tables and figures**

**Additional Table 1** **Characteristics of the study population involved in the assessment of exposure to *Anopheles*’ bite**

|  |  |  |  |
| --- | --- | --- | --- |
|  |  | Kwamasimba (n=1296) | Mkokola (n=1266) |
| Gender, n (%) | Female | 715 (55.2) | 673 (53.2) |
|  | Male | 580 (44.8) | 592 (46.8) |
| Malaria prevalence by blood smear, n (%) | Negative | 1,200 (93.4) | 1,011 (80.4) |
|  | Positive | 85 (6.6) | 247 (19.6) |
| Bed net use, n (%) | No | 395 (30.7) | 246 (19.5) |
|  | Yes | 892 (69.3) | 1016 (80.5) |
| Age groups, n (%) | 0-4 | 466 (36.0) | 391 (30.9) |
|  | 5-9 | 444 (34.3) | 415 (32.8) |
|  | 10-14 | 252 (19.4) | 328 (25.9) |
|  | 15-19 | 134 (10.3) | 132 (10.4) |
| Year of survey, n (%) | 2004 | 80 (6.2) | 79 (6.2) |
|  | 2005 | 120 (9.3) | 105 (8.3) |
|  | 2006 | 104 (8.0) | 108 (8.5) |
|  | 2007 | 99 (7.6) | 97 (7.7) |
|  | 2008 | 99 (7.6) | 77 (6.1) |
|  | 2012 | 94 (7.3) | 90 (7.1) |
|  | 2014 | 100 (7.7) | 99 (7.8) |
|  | 2015 | 102 (7.9) | 99 (7.8) |
|  | 2016 | 100 (7.7) | 106 (8.4) |
|  | 2017 | 100 (7.7) | 104 (8.2) |
|  | 2018 | 100 (7.7) | 104 (8.2) |
|  | 2019 | 98 (7.6) | 99 (7.8) |
|  | 2021 | 100 (7.7) | 99 (7.8) |


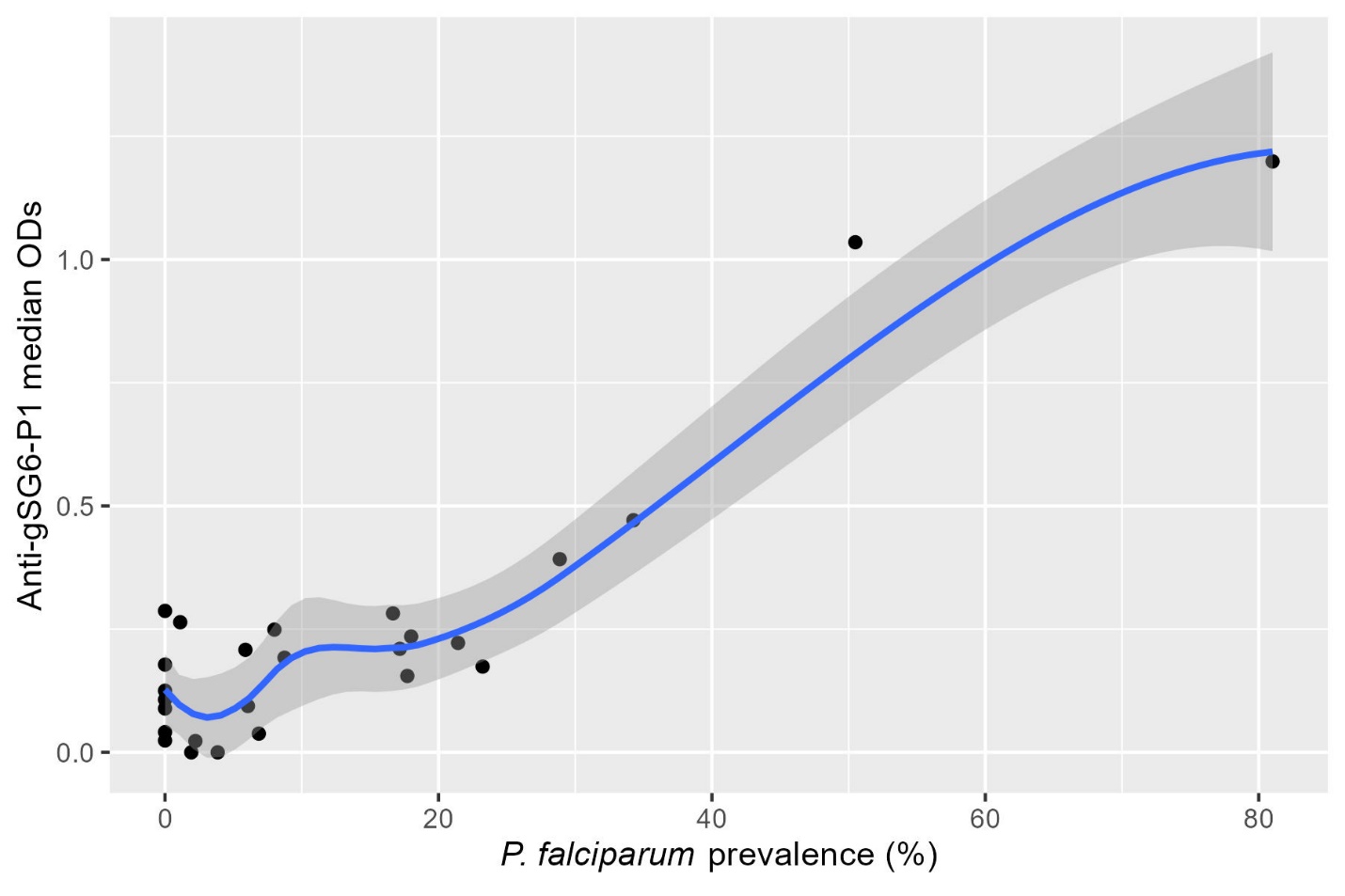


**Additional Figure 1** The malaria point prevalence and anti-gSG6-P1 antibody levels in Kwamasimba and Mkokola showed a strong correlation (Spearman’s rho = 0.6, *p* < 0.001).

**Additional table 2. Seasonal variation in *P. falciparum* prevalence and bed net use in Kwamasimba and Mkokola villages.** A: Study population involved in all 18 malariometric cross-sectional survey, Kwamasimba n = 4915 and Mkokola n = 4921. B: Sub-population from 13 of the 18 cross-sectional survey that was investigated for *Anopheles’* bite exposure, Kwamasimba n = 1296 and Mkokola n = 1266.

|  |  | | | |  | | | |
| --- | --- | --- | --- | --- | --- | --- | --- | --- |
|  | Kwamasimba | | Mkokola | | Kwamasimba | | Mkokola | |
| Year | *P. falciparum*  prevalence (%) | Bed net  use (%) | *P. falciparum*  prevalence (%) | Bed net  use (%) | *P. falciparum*  prevalence (%) | Bed net  use (%) | *P. falciparum*  prevalence (%) | Bed net  use (%) |
| 2003 | 24.7 | 3.5 | 78.6 | 32.0 | *NA** | *NA** | *NA** | *NA** |
| 2004 | 20.2 | 28.2 | 77.4 | 43.3 | 17.7 | 32.5 | 81.0 | 35.9 |
| 2005 | 7.8 | 38.5 | 52.2 | 48.5 | 5.9 | 29.4 | 50.5 | 59.1 |
| 2006 | 2.8 | 57.2 | 30.6 | 63.3 | 1.9 | 60.2 | 34.3 | 69.4 |
| 2007 | 7.6 | 63.4 | 24.2 | 69.3 | 6.1 | 65.0 | 28.9 | 77.1 |
| 2008 | 3.2 | 45.5 | 12.2 | 71.1 | 2.2 | 49.0 | 16.7 | 72.7 |
| 2009 | 0.4 | 68.3 | 9.7 | 82.7 | *NA** | *NA** | *NA** | *NA** |
| 2010 | 0.4 | 50.6 | 5.3 | 82.8 | *NA** | *NA** | *NA** | *NA** |
| 2011 | 0.00 | 49.8 | 1.1 | 68.4 | *NA** | *NA** | *NA** | *NA** |
| 2012 | 0.4 | 83.9 | 2.3 | 95.4 | 0.0 | 80.7 | 1.1 | 94.4 |
| 2013 | 0.0 | 88.0 | 7.6 | 95.9 | *NA** | *NA** | *NA** | *NA** |
| 2014 | 0.0 | 77.4 | 0.0 | 93.0 | 0.0 | 82.0 | 0.0 | 90.9 |
| 2015 | 5.0 | 56.0 | 23.9 | 80.9 | 6.9 | 56.9 | 23.2 | 78.8 |
| 2016 | 0.0 | 98.6 | 1.2 | 100.0 | 0.0 | 100.0 | 0.0 | 100.0 |
| 2017 | 0.3 | 94.8 | 4.8 | 99.6 | 0.0 | 91.0 | 3.9 | 99.0 |
| 2018 | 17.5 | 88.2 | 10.3 | 85.6 | 18.0 | 86.0 | 8.7 | 82.7 |
| 2019 | 16.8 | 77.6 | 18.4 | 78.9 | 21.4 | 79.0 | 17.2 | 76.8 |
| 2020 | *NA^#^* | *NA^#^* | *NA^#^* | *NA^#^* | *NA^#^* | *NA^#^* | *NA^#^* | *NA^#^* |
| 2021 | 7.9 | 93.3 | 2.1 | 99.4 | 8.0 | 91.0 | 0.0 | 100.0 |

*NA* Samples were not available for Anopheles’ bite exposure, NA^#^ in 2020 cross-sectional surveys were not conducted.*

**Additional Table 3 Univariate and multivariate linear regression results showing the association between anti-gSG6-P1 and gender, bed net use and age groups in the Kwamasimba highland village.**

|  |  | Coef.  [95% Conf. Interval] | p-value | | Adjusted Coef.  [95% Conf. Interval] | Adjusted p-value | Overall adjusted *p-*value |
| --- | --- | --- | --- | --- | --- | --- | --- |
| Gender | Female | Ref | Ref | | Ref | Ref | 0.001* |
|  | Male | 0.009 (-0.038 – 0.057) | 0.700 | | 0.027 (-0.021 – 0.074) | 0.270 |  |
|  |  |  |  | |  |  |  |
| Bed net use | No | Ref | Ref | | Ref | Ref |  |
|  | Yes | -0.080 (-0.132 – -0.029) | 0.002* | | -0.073 (-0.123 – -0.022) | 0.005* |  |
|  |  |  |  | |  |  |  |
| Age groups | 0 – 4 | Ref | Ref | | Ref | Ref |  |
|  | 5 – 9 | 0.095 (0.039 – 0.150) | 0.001* | 0.001* | 0.093 (0.038 – 0.149) | 0.001* |  |
|  | 10 – 14 | 0.232 (0.166 – 0.297) | 0.001* |  | 0.233 (0.167 – 0.299) | 0.001* |  |
|  | 15 – 19 | 0.256 (0.174 – 0.337) | 0.001* |  | 0.250 (0.168 – 0.333) | 0.001* |  |

Coef. Coefficients, *Statistical significance.

**Additional Table 4 Univariate and multivariate linear regression results showing the association between anti-gSG6-P1 and gender, bed net use and age groups in the Mkokola lowland village.**

|  |  | Coef (95% Conf. Interval) | p-value | | Adjusted Coef. (95% Conf. Interval) | Adjusted p-value | Overall adjusted *p-*value |
| --- | --- | --- | --- | --- | --- | --- | --- |
| Gender | Female | Ref | Ref | | Ref | Ref | 0.001 |
|  | Male | -0.008 (-0.068 – 0.051) | 0.784 | | 0.007 (-0.052 – 0.066) | 0.809 |  |
|  |  |  |  | |  |  |  |
| Bed net use | No | Ref | Ref | | Ref | Ref |  |
|  | Yes | -0.337 (-0.410 – -0.264) | 0.001* | | -0.330 (-0.403 – -0.256) | 0.001* |  |
|  |  |  |  | |  |  |  |
| Age groups | 0 – 4 | Ref | Ref | | Ref | Ref |  |
|  | 5 – 9 | 0.201 (0.128 – 0.274) | 0.001* | 0.001* | 0.173 (0.098 – 0.248) | 0.001* |  |
|  | 10 – 14 | 0.261 (0.184 – -0.339) | 0.001* |  | 0.239 (0.159 – 0.318) | 0.001* |  |
|  | 15 – 19 | 0.287 (0.182 – 0.391) | 0.001* |  | 0.264 (0.159 – 0.369) | 0.001* |  |

Coef. Coefficients, *Statistical significance.
